# A Case of Supplement-Induced Hepatotoxicity

**DOI:** 10.1155/2010/262706

**Published:** 2010-08-15

**Authors:** Fong-Kuei Frank Cheng, Peter Dunaway

**Affiliations:** ^1^Department of Medicine, Madigan Army Medical Center, Tacoma, WA 98431, USA; ^2^Gastroenterology Service, Department of Medicine, Madigan Army Medical Center, Tacoma, WA 98431, USA

## Abstract

A 45-year-old Caucasian male presented with a two-week history of jaundice and right-upper quadrant (RUQ) abdominal pain. Transaminases and biliary enzymes were markedly elevated with hyperferritinemia and mildly elevated INR. Imaging tests showed no significant abnormality. He denied prescription or over-the-counter (OTC) medication use, but he had been taking at least 9 dietary supplements for 12 months. Other causes of liver disease were excluded. His supplements were discontinued, and his liver-associated enzymes significantly markedly improved over the next 6 weeks and remained normal after one year suggesting supplement-induced hepatotoxicity. Due to the number of supplements, no specific agent could be identified as the primary cause of his liver injury. This case illustrates the importance of inquiring and educating patients of the potential harmful risks of over-the-counter medications and supplements.

## 1. Introduction

Over-the-counter (OTC) supplement use has increased in the United States over the past decade, and sources estimate that consumers can now choose from over 29,000 products [1]. Dietary supplement-related hepatotoxicity, an underreported condition, is usually associated with minor liver-associated enzyme elevations [2]. We describe a case of severe hepatotoxicity with jaundice and mild synthetic dysfunction in a patient taking multiple dietary supplements. 

## 2. Case

A previously healthy 45-year-old white male complained of dull aching RUQ pain associated with nausea two weeks prior to his initial presentation to primary care. The pain was constant, nonradiating, and most intense two hours after meals. The pain was followed by icterus, jaundice, and generalized weakness, which progressively worsened over the week. He had secondary anorexia over the symptomatic period and had lost 20 lbs over the previous two months. He denied pruritus, easy bleeding/bruising, melena, abdominal distention, mental status changes, increased somnolence, diarrhea, or fever.

He initially reported taking only a few daily supplements over the previous 12 months for the promotion of general health. However, on further questioning, he admitted using nine different products many of which had been started one-to-four months prior to his symptom onset (Table 1). He had stopped all supplements intake after the development of his symptoms.

Additional risk factors for secondary causes of liver disease were negative to include a history of viral hepatitis, blood product transfusion, intravenous drug abuse, multiple sexual partners, and prescription medications. He consumed two-to-three mixed drinks on social occasions and was not a habitual alcohol consumer. He denied acetaminophen use or the ingestion of wild mushrooms. He recently vacationed with his wife in Puerto Rico three months prior to presentation but denied any additional foreign travel. He had no family history of liver disease and denied any ill contacts.

His physical examination was only notable for jaundice, scleral icterus, and mild RUQ abdominal tenderness on deep palpation. Significant presentation labs are as follows: ALT 6409 U/l, AST 3505 U/l, alkaline phosphatase 269 U/L, total bilirubin 31 mg/uL, conjugated bilirubin 18 mg/dL, prothrombin time 12.6 sec, and INR 1.2. Iron studies were ferritin 2641 ng/mL, fasting iron 235 ug/dL, TIBC 290 ug/dL, and % transferrin sat 81%. CBC was normal without eosinophilia. Serum acetaminophen level was immeasurable as the severe hyperbilirubinemia interfered with the assay. The patient did not have any baseline liver enzymes for comparison. RUQ ultrasound and CT abdomen revealed mild diffuse thickening of the gallbladder without cholelithiasis or intra/extrahepatic ductal dilatation. The pancreas, liver, portal veins, and spleen were otherwise normal. 

**Figure 1:**
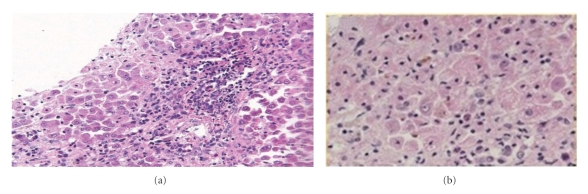
(a) Periportal triad inflammation (b) cholestasis and hepatocyte necrosis (zone 3) iron liver 321 ug/g dry wt (400–2200), hepatic iron index 0.1 umoL/g/yr (<1.0).

The workup for other causes of liver disease includes IgM anti-HAV, IgM anti-HBc, HbsAg, anti-HCV, ceruloplasmin, mitochondrial antibody, ANA, SPEP, and anti-LKM which were negative/normal except for the presence of one heterozygous copy of the H63D mutation in the HFE gene and a highly positive smooth muscle antibody of 32 (>30 highly positive). Liver histopathology revealed mild periportal and intraparenchymal chronic inflammation, including plasma cells, with focal areas of acute inflammation and patchy hepatocyte necrosis with moderately increased iron storage (Iron liver dry weight 321 ug/g (400–2200) hepatic iron index 0.1 umoL/g/yr (<1.0)) (Figure 1). Copper studies of liver biopsy were not performed. 

**Figure 2:**
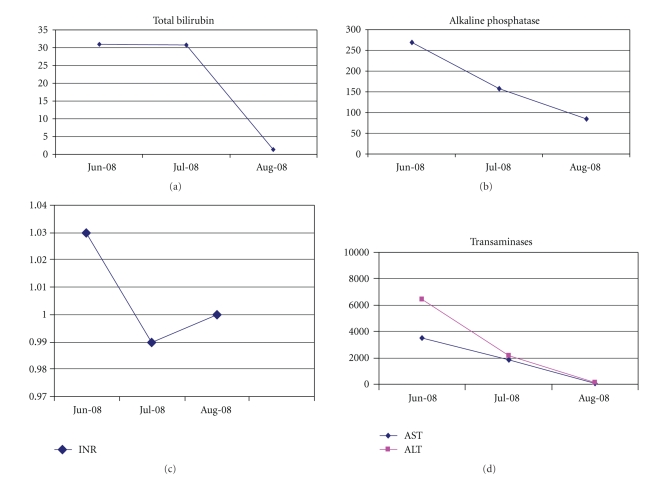
Followup labs at 6 weeks.

The liver biopsy was secondarily reviewed by the Armed Forces Institute of Pathology that reported widespread zone 3 (centrilobular) confluent necrosis and stromal collapse with patchy portal inflammation and considerable bile stasis. Their interpretation of the combined cholestatic and hepatocellular injury pattern was consistent with drug/supplement-related hepatotoxicity. The findings were not consistent with autoimmune hepatitis. At 6 weeks, the patient's symptoms had resolved. Repeat liver-associated enzymes had nearly normalized (ALT-135 U/L and total bilirubin-1.4 mg/uL) and iron studies showed a % iron saturation of 52% and serum ferritin of 196 ng/ml (Figure 2). At one year, his liver enzymes were completely normal. To date, the patient remains asymptomatic and continues to avoid OTC supplements.

## 3. Discussion

Approximately 65% of the US population reports use of complementary and alternative medical (CAM) therapies. In 1997, US consumers spent $46 billion on CAM therapies and dietary supplements. Women and non-Hispanic whites more frequently use alternative medicines compared to males and other racial groups, respectively [1]. While many explanations have been offered for increased use of CAM therapy, users generally view natural products as superior and innocuous compared to traditional medications. These consumers also often seek alternative treatments when they are not satisfied with conventional treatment [3].

In 1994, the Dietary Supplement Health and Education Act expanded the responsibilities of the Food and Drug Administration (FDA) to include monitoring safety and product information of CAM therapies [4]. Enforcement of this regulation has proved problematic, as manufacturers are still not required to register for FDA approval or prove a product's safety prior to marketing [4]. Content labeling and product consistency remain variable, and adverse effects from these products are likely underreported. 

Hepatotoxicity is common with both CAM and conventional medication therapies, due to the liver's primary role in drug metabolism [2]. Medication-induced liver injury is often grouped into types of injury based on biochemical and histological abnormalities, and certain medications have been classically associated with specific injury patterns [5]. Our patient manifested a mixed liver injury pattern. Our case is interesting in that our patient's transaminases were three-to-six times higher than those of other cases reported in the medical literature [6]. The clinical and laboratory presentation represents severe hepatotoxicity with mild synthetic dysfunction and rapid resolution, while many cases in literature progress to acute liver failure.

Our patient was taking nine different supplements which comprised of over thirty individual ingredients (Tables 1 and 2). He was also taking several supplements which contained no ingredient description. All available ingredients were reviewed for potential harm using the original product's manufacturer website, the consumer reports from the US Food and Drug Administration, and annual reports from the American Association of Poison Control Center. Table 3 lists common herbal remedies associated with hepatotoxicity [7, 8]. When comparing the patient's list of ingredients to the common toxins causing hepatotoxicity, potential offending agents include vitamin A, copper, iron, and niacin. Unfortunately a specific contributing source could not be ascertained, due to the large number of supplements involved and the unknown ingredients of a few of the supplements. Our patient's markedly elevated ferritin and antismooth muscle antibodies were most likely due to the supplement-induced acute liver injury. His increased % iron saturation, interestingly, is secondary to his H63D heterozygous mutation as his ferritin was normalized in followup but the increased % iron saturation persisted. The low hepatic iron weight and hepatic iron index ruled out hemochromatosis. Autoimmune hepatitis was ruled out clinically and confirmed by the normalization of his liver enzymes.

Treatment for supplement-induced hepatotoxicity consists of prompt cessation of all supplement use. The development of any encephalopathy or worsening of coagulopathy requires an immediate referral to a medical center with a liver transplantation service [9]. There are no confirmatory tests; however, appropriate evaluation to exclude other underlying hereditary or acquired liver disease is prudent. Clinical improvement occurs in most cases after discontinuance of offending agent, but liver injury may worsen or follow a protracted recovery course of weeks or months [9]. If patients develop acute fulminant hepatic failure, important prognostic indicators include the degree of encephalopathy, the patient's age, prothrombin time, and the cause of acute liver failure [9]. Given the absence of encephalopathy, prompt normalization of his initial mild protime elevation, and gradual recovery within weeks after discontinuing his supplements, liver transplantation was not considered in our patient. This case emphasizes the importance of recognizing the widespread use of over-the-counter supplements and herbal remedies. Health care providers should inform their patients about the potentially harmful risks of over-the-counter supplement use, particularly hepatotoxicity. Providers should also be vigilant about reporting any suspected supplement-related hepatotoxicity to the FDA immediately.

## Figures and Tables

**Table 1:** Reported supplements in this case.

*Mixture of ingredients without dosage listed	
(1) Living Multi * Optimal Men's Formula * Vitamin A, vitamin C, vitamin D, vitamin E, thiamine, riboflavin, niacin, vitamin B6, folic acid, vitamin B12, biotin, pantothenic acid, calcium, iodine, iron, magnesium, zinc, selenium, copper, manganese, chromium, molybdenum, potassium, boron, vanadium, choline, lycopene, lutein (Natural Antioxidant Fruit and Veggie Blend, Poten-Zyme Sea Veggie Blend, Men's Blend, Poten-Zyme Tonic Mushroom Blend, Poten-Zyme Veggie Blend, Antioxidant Beverage and Spice Blend) http://www.gardenoflifeusa.com/ProductsforLife/SUPPLEMENTS/LivingNutrients/LivingMultiMens/tabid/668/Default.aspx 4 per day in morning with breakfast	

(2) Living Calcium Advanced Bone Density Support Formula* Vitamin D, riboflavin, vitamin B6, folic acid, vitamin B12, calcium, iron, magnesium, zinc, copper, manganese, boron, vitamin K, betaine (Bone Support Blend, Vital Mineral Matrix, Homocysteine Balance Complex) http://www.gardenoflifeusa.com/ProductsforLife/SUPPLEMENTS/LivingNutrients/LivingCalciumAdvanced/tabid/666/Default.aspx 3 per day in morning for broken elbow	

(3) Perfect Food *Super Green Formula Caplets * Vitamin A, vitamin C, calcium, iron (Poten-Zyme Whole Food Matrix, Perfect Green Juice Blend, Perfect Protein Mineral Blend, Acerola Cherry Extract, Perfect Veggie Juice Blend, Probiotic Blend) http://www.gardenoflifeusa.com/ProductsforLife/SUPPLEMENTS/FoundationalNutrition/PerfectFood/tabid/654/Default.aspx 2 per day in morning	

(4) FYI ULTRA *Ultimate Joint & Cartilage Formula * Selenium, glucosamine HCL (Protective Tissue Response Blend, Antioxidant Cartilage Health Complex) http://www.gardenoflifeusa.com/ProductsforLife/SUPPLEMENTS/ImmunitySupport/FYIULTRA/tabid/658/Default.aspx 3 per day in morning	

(5) *füco*Thin *Concentrated fucoxanthin * (Xanthigen Proprietary Blend) http://www.fucothin.com/FAQs/tabid/1160/Default.aspx 1 in morning and 1 in evening with meal “dietary supplement”	

(6) Bone Strength Take Care Vitamin D, calcium, magnesium, vanadium, vitamin K, strontium, silica, betaine http://www.newchapter.com/products/bone-strength-take-care 2 per day in evening for broken elbow	

(7) 4Total Nutrition Individual ingredients not listed http://www.genesistoday.com/genesis_today_products/4total_nutrition.html 1 tablespoon mixed with juice in evening (stopped taking after 2 weeks, bad taste)	

(8) POWER4 Individual ingredients not listed http://www.genesistoday.com/genesis_today_products/power_4.html 1 tablespoon mixed with juice in morning “use this to drink down my vitamins”	

(9) Ab-Solution Plus Individual ingredients not listed http://www.vyotech.com/products/absolutionplus/ Rub on stomach area once in the morning and in the evening	

**Table 2:** Summary of individual ingredients in supplements that exceeded recommended daily allowance (RDA) in this case.

	RDA%	RDA	Total
Vitamin A (IU)	416.67%	3000	12,500
Vitamin C (mg)	166.67%	90	150
Vitamin D (IU)	900.00%	200	1,800
Vitamin E (IU)	200.89%	22.4	45
Thiamine (B1) (mg)	375.00%	1.2	5
Riboflavin (B2) (mg)	1161.54%	1.3	15
Niacin (B3) (mg)	187.50%	16	30
Vitamin B6 (mg)	2384.62%	1.3	31
Folic acid (mcg)	200.00%	400	800
Vitamin B12 (mcg)	10333.33%	2.4	248
Biotin (mcg)	3000.00%	30	900
Pantothenic Acid (mg)	600.00%	5	30
Calcium (mg)	208.00%	1000	2,080
Iodine (mcg)	120.00%	150	180
Magnesium (mg)	153.10%	420	643
Zinc (mg)	163.64%	11	18
Selenium (mcg)	363.64%	55	200
Manganese (mg)	173.91%	2.3	4
Chromium (mcg)	571.43%	35	200
Molybdenum (mcg)	166.67%	45	75
Vitamin K (mcg)	170.83%	120	205

**Table 3:** Common herbal supplements associated with hepatotoxicity.

Herbal supplement induced hepatotoxicity	
Pyrrolizidine alkaloids	Ma-Huang
Germander	Syo-saiko-to
Greater celandine	Bajiaolian
Chaparral	Borage
Atractylis gummifera	Broom corn
Pennyroyal	Callilepis
Mistletoe	Margosa Oil
Kava Kava	LipoKinetix
Camellia sinensis	Hydroxycut
Jin Bu Huan	HerbaLife
